# Reconciling taxonomy and phylogenetic inference: formalism and algorithms for describing discord and inferring taxonomic roots

**DOI:** 10.1186/1748-7188-7-8

**Published:** 2012-05-02

**Authors:** Frederick A Matsen, Aaron Gallagher

**Affiliations:** 1 Fred Hutchinson Cancer Research Center, Seattle, Washington, USA

**Keywords:** phylogenetics, taxononomy, dynamic program, branch and bound, convex coloring, algorithms

## Abstract

**Background:**

Although taxonomy is often used informally to evaluate the results of phylogenetic inference and the root of phylogenetic trees, algorithmic methods to do so are lacking.

**Results:**

In this paper we formalize these procedures and develop algorithms to solve the relevant problems. In particular, we introduce a new algorithm that solves a "subcoloring" problem to express the difference between a taxonomy and a phylogeny at a given rank. This algorithm improves upon the current best algorithm in terms of asymptotic complexity for the parameter regime of interest; we also describe a branch-and-bound algorithm that saves orders of magnitude in computation on real data sets. We also develop a formalism and an algorithm for rooting phylogenetic trees according to a taxonomy.

**Conclusions:**

The algorithms in this paper, and the associated freely-available software, will help biologists better use and understand taxonomically labeled phylogenetic trees.

## Background

Since the beginnings of phylogenetics, researchers have used a combination of phylogenetic inference and taxonomic knowledge to understand evolutionary relationships. Taxonomic classifications are often used to diagnose problems with phylogenetic inferences, and conversely, phylogeny is used to bring taxonomies up to date with recent inferences and to find misclassified sequences. Similarly, biologists often evaluate a putative "root" of a phylogenetic tree by looking at the taxonomic classifications of the subtrees branching off that node.

Despite the long history of interaction between phylogeny and taxonomy, automated tools for the automated curation of taxonomies have only recently been developed. In 2007, Dalevi *et. al. *[1] released the GRUNT tool to refine existing taxonomic classifications and propose novel ones. This was followed just recently by McDonald *et. al. *[2], who developed the tax2tree tool to update taxonomies based on a measure of precision and recall for classifications. These tools aim to update taxonomies to be closer to phylogenetic inferences.

In this paper we approach the commonly encountered simpler problem of a researcher inferring a phylogenetic tree and wishing to understand the level of agreement of that tree with a taxonomy at various ranks and wishing to root the tree taxonomically. We state the agreement problem between a taxonomy and a phylogeny in terms of an "subcoloring" problem previously described in the computer science literature [3,4]. As described below, we make algorithmic improvements over previous work in the relevant parameter regime and present the first computer implementation to solve the subcoloring problem. Our choice of algorithms is guided by the parameter regime of relevance for modern molecular phylogenetics on marker genes: that of large bifurcating trees and a limited amount of discord with a taxonomy. For rooting, we show that the "obvious" definition has major defects when there is discordance between a phylogeny and a taxonomy at the highest multiply-occupied taxonomic rank. We then present a more robust alternative definition and algorithms that can quickly find a taxonomically-defined root.

We emphasize that our work is in describing discordance between a taxonomy and a phylogeny and performing taxonomic rooting rather than updating taxonomies, in contrast to the work described above. However, our approach to expressing discord between a taxonomy and a phylogeny can be used to suggest that certain sequences are misclassified; we will explore this direction in future work.

## Expressing the differences between a taxonomy and a phylogeny

### Informal introduction

In this paper we will consider agreement with a taxonomy one taxonomic rank at a time, in order to separate out the different factors that can lead to discord between taxonomy and phylogeny. These factors include phylogenetic methodology problems, out of date taxonomic hierarchies, and mislabeling. Various such factors lead to discordance at distinct ranks. For example, we have observed rampant mislabeling at the species level in public databases, whereas higher-level assignments are typically more accurate. Phylogenetic signal saturation or model mis-specification problems can lead to an incorrect branching pattern near the root of the tree at the higher taxonomic levels, although the genus-level reconstructions can be correct.

An alternative to considering agreement one rank at a time would be to look for the largest set of taxa for which the induced taxonomy and phylogenetic tree agree on all levels. Agreement between taxonomy and phylogeny at all taxonomic ranks simultaneously is equivalent to requiring complete agreement of a phylogeny and a taxonomic tree. Finding a subset of leaves on which two trees agree is known as the Maximum Compatible Subtree (MCST) problem, known to be polynomial for trees of bounded degree and NP-hard otherwise [5]. Although such a solution is useful information, we have pursued a rank-by-rank approach here for the reasons described above.

**Figure 1:**
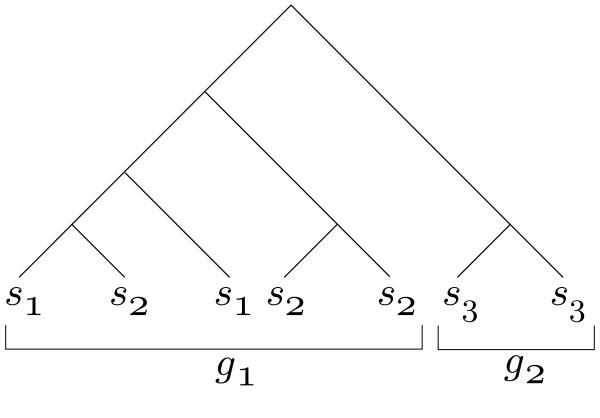
**Coloring and taxonomic assignments**. A taxonomically labeled phylogenetic tree that is concordant with the genus level taxonomic assignments *g_i _*but not the species taxonomic assignments *s_i_*.

We formalize the agreement of a taxonomy with a phylogeny on a rank-by-rank basis in terms of *convex colorings *[3,4]. Informally, a convex coloring is an assignment of the leaves of a tree to elements of a set called "colors" such that the induced subtrees for each color are disjoint. In this paper we will say that a phylogeny agrees with taxonomic classifications at taxonomic rank *r *if the taxonomic classifications at rank *r *induce a convex coloring of the tree. For example, in Figure 1 the tree is not convex at the species rank due to nonconvexity between species *s*_1 _and *s*_2_, although it is convex at the genus rank, as the *g*_1 _and *g*_2 _genera fall into distinct clades. In terms of the convex coloring definition, there is nontrivial overlap between the induced trees on *s*_1 _and *s*_2_.

We will express the level of agreement between a taxonomy and a phylogeny at a rank *r *in terms of the size of a *maximal convex subcoloring*. Given an arbitrary leaf coloring, a subcoloring is a coloring of some subset of the leaves *S *of the tree that agrees with the original coloring on the set *S*. The maximal convex subcoloring is the convex subcoloring of maximal cardinality. For a tree that is taxonomically labeled at the tips, the discord at rank *r *is defined to be the size of the maximal convex subcoloring when the leaves are colored according to the taxonomic classifications at rank *r*.

Our algorithmic contributions result in efficient algorithms for the convex subcoloring problem in the parameter regime of interest: a limited amount of discord for large trees. First, by developing an algorithm that only investigates removing colors when such a removal could make a difference, we show that the maximal convex subcoloring problem can be solved in a number of steps that scales in terms of a local measure of nonconvexity rather than the total number of nonconvex colors. Second, we implement a branch and bound algorithm that terminates exploration early; this algorithm makes orders of magnitude improvement in run times for difficult problems.

**Figure 2:**
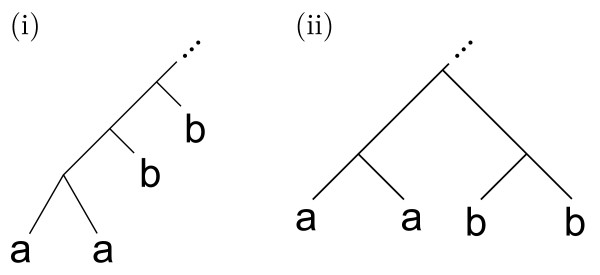
**An alternative definition of convexity**. Example colored trees showing the (i) original and (ii) strong definition of convexity, assuming a and b don't appear elsewhere in the tree. In this figure, (i) and (ii) are convex according to the original definition, but only (ii) is convex according to the strong definition.

Before proceeding on to outline how the algorithm works, note that the original definition of convexity is not the only way to formalize the agreement with a taxonomy at a given rank: a stronger way of defining convexity is possible (Figure 2). In this "strong" version, colors must sit in disjoint rooted subtrees rather than just in disjoint induced subtrees. The algorithmic solution for this stronger version will be a special case of the previous one as described below. It may be of more limited use for two reasons. First, it depends on the position of the root: a tree that is strongly convex with one rooting may not be so in another. Also, it is not uncommon for phylogenetic algorithms to return a tree like in Figure 2(ii) although Figure 2(i) may actually be the correct tree; thus an algorithm that threw out all leaves except those that are convex in the strong sense might be overly strict.

**Figure 3:**
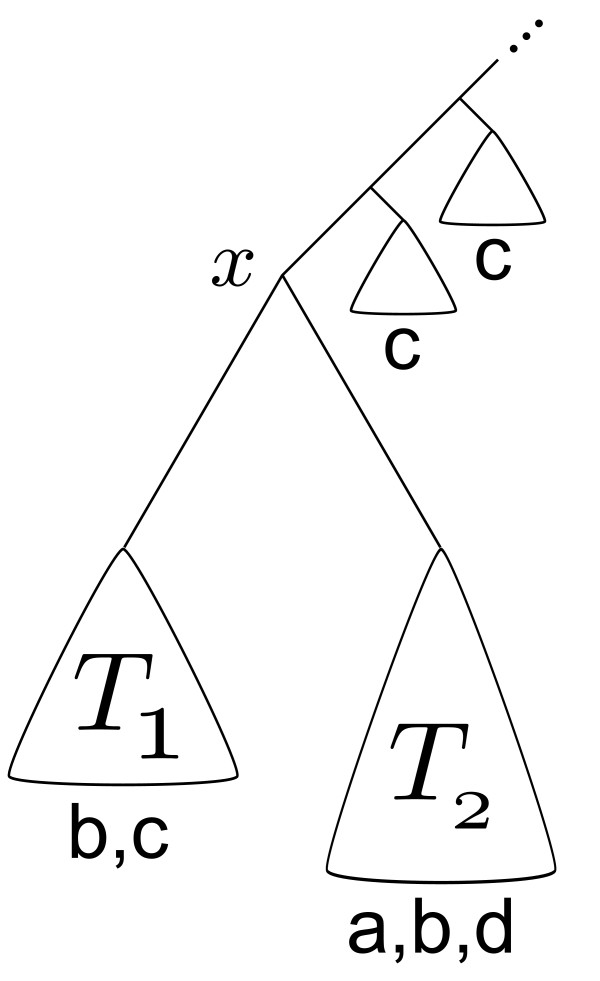
**Recursion example**. A possible scenario encountered by the subcoloring recursion. The letters a, b, c and d represent the presence of leaves with those taxonomic labels; asssume these taxonomic labels do not occur elsewhere in the tree. The positions of colors b and c shows that this coloring is not convex, and a recursive subcoloring algorithm must decide at *x *in which subtrees to allow the b and c colors.

The tree in Figure 3 serves to explain why the problem is combinatorially complex and motivates a recursive solution. The idea of this solution is to recursively descend through subtrees, starting at the root. Say this recursion has descended to an internal node *x*, and there are nodes of the color c somewhere above *x*. If the set of leaf colors in the subtree *T*_1 _is {b, c} and if the set of leaf colors in the subtree *T*_2 _is {a, b, d}, then some removal of colors is needed due to nonconvexity between the b and c colors. Assuming the leaf colors above *x *are fixed, the choices are to uncolor the c nodes in *T*_1 _or the b nodes in either *T*_1 _or *T*_2_.

One can think of "allocating" the cut colors to the subtrees: the possible choices are to allocate c to *T*_1 _but choose one of *T*_1 _or *T*_2 _to have b, or to disallow c in *T*_1 _and allow b in both *T*_1 _and *T*_2_. Here and in general, the crux of devising an efficient recursion is to efficiently decide which colors get allocated to which subtrees. Convexity can be insured by explicitly choosing a color for each internal node, and making sure that the color allocations respect those internal node choices in terms of convexity.

In fact, selecting these color allocations is the only problem, as a complete set of color allocations is trivially equivalent to a choice of coloring. Indeed, given an optimal color allocation for each internal node, one can simply look at the allocations for the internal nodes just above the leaves to decide whether those leaves get uncolored or not. Conversely, given a leaf coloring, one can simply look at the color set of the descendants of that internal node to get the set of colors allocated to the subtrees.

In deciding the color allocations, we can restrict our serious attention to colors that are present on either side of an edge, such as b and c on either side of the edge directly above the root of *T*_1 _in Figure 3. We will say that these colors are *cut *by the edge. Colors that are not cut by an edge should not require any decision making when the recursive algorithm is visiting the node just above that edge. However, doing the accounting is not completely straightforward: of the cut colors, one might only allocate b to *T*_2_, but a and d can be used as well. Thus, allocations including some colors not cut by the current edge must be considered, motivating the definition of the *colors in play *(Definition 10). The colors in play for an internal node are those colors cut by edges directly below that node that are available for allocation to the trees below that internal node.

Note that the ingredients of the decision being made in Figure 3 are the color specified by the coloring just above *x *(in this case fixed to be c), and the colors available in the subtrees below *x*. Given a set of colors to allocate to the leaves below *x*, the algorithm needs to decide how to allocate the possible colors to *T*_1 _and *T*_2_. One way of doing that is to test every possible allocation using the results of the recursion for below subtrees and score them in terms of the size of the corresponding subcoloring. Doing this with an awareness of the cut colors leads to an algorithm expressed in terms of the maximum number of colors cut by a given edge.

However, building such a comprehensive optimality map is not necessary. By simply counting the number of leaves of each color below *x*, one can get upper bounds on the sizes of the corresponding subcolorings and only evaluate those that have the potential to be worth exploring. This observation is the basis of the branch and bound algorithm (Algorithm 1).

### Definitions and algorithms

A *rooted subtree *is a subtree that can be obtained from a rooted tree *T *by removing an edge of *T *and taking the component that does not contain the original root of *T*. The *proximal *direction in a rooted tree is towards the root, while the *distal *direction is away from the root. Given a tree *T *, let *N(T), E(T)*, and *L(T) *denote the nodes, edges, and leaves of *T*. Given a set *U*, let 2*^U ^*denote the set of subsets of *U*. If the input tree to the algorithm is not rooted, root it arbitrarily. Following the terminology of [3,4], a *color set *will be an arbitrary finite set, always denoted in this paper by the letter *C*.

**Definition 1**. *Let T be a rooted tree, and let F *⊆ *L (T)*. A leaf coloring *is a map χ*: *F *→ *C*.

A color *c *is *cut *by an edge *e *if there is at least one leaf of color *c *on either side of *e*. A *multicoloring *is defined to be a map from the edges of the tree to subsets of the colors.

**Definition 2**. *Given a coloring χ on a rooted tree T, the *induced multicoloring *for χ is the map *$\tilde{\chi }:E\left(T\right)\to 2^{C}$*such that *$\tilde{\chi }\left(e\right)$*is the (possibly empty) set of colors cut by that edge*.

**Definition 3**. *Define the *badness *β of a coloring χ to be *${\text{max}}_{e\in E\left(T\right)}\left|\tilde{\chi }\left(e\right)\right|$. *We say that a coloring is *convex *if it has badness equal to zero or one*.

**Definition 4**. *The *total number of bad colors *of a coloring χ is *$\tau \;=\;\left|\cup_{e\in E}\;\tilde{\chi }\left(e\right)\right|$*where *E *is the set of edges where *$\left|\tilde{\chi }\left(e\right)\right|\;\geq \;2$.

**Definition 5**. *A subcoloring of a leaf coloring χ*: *F *→ *C is a coloring χ': G *→ *C with G *⊆ *F such that χ' agrees with χ on the domain of χ'*.

Subcolorings are partially ordered by inclusion of domains; the size of a subcoloring is defined to be the size of its domain.

**Problem 1 **(Moran and Snir [3,4]). *Given a leaf coloring χ on a tree T, find a largest convex subcoloring*.

#### Previous work and motivation for present algorithm

The foundational work in this area was done by Moran and Snir [3,4]. Their work is phrased in terms of "convex recoloring," i.e. finding the minimal number of changes in a coloring in order to obtain one that is convex.

It suffices to consider subcolorings for the case of leaf colorings. Indeed, any recoloring can be turned into a subcoloring by removing the color of all of the leaves that get recolored. Conversely, any convex subcoloring can be turned into a convex recoloring in linear time [6]. For internal nodes, the color to be used for a given internal node is given by the definition of convex coloring. For leaf nodes, simply take the color of the closest colored node. In this equivalence, the number of leaves whose color is removed is equal to the number of leaves who get recolored; thus a minimal recoloring is equivalent to a maximal subcoloring. Because of this equivalence, we only consider subcolorings in this paper.

In [4], Moran and Snir investigate both the general case of leaf colorings as well as the case of colorings including internal nodes. They also consider non-uniform recoloring cost functions, where a "cost" is associated with recoloring individual nodes and the goal is to find a convex recoloring minimizing total cost. In all settings, they demonstrate that the relevant recoloring problem is NP-hard. They also demonstrate fixed parameter tractablity (FPT) of the problems as described in the next paragraph. In [3] they present, among other results, a 3-approximation for tree recoloring.

The FPT bound for leaf coloring an *n *taxon tree from [4], *O*(*n*^4 ^*τ *Bell(*τ*)), comes from an elegant argument using the Hungarian algorithm for maximum weight perfect matching on a bipartite graph. In fact, an inspection of their proof reveals that their algorithm is *O*(*n d*^3 ^*τ *Bell(*τ*)), where *d *is the maximum degree of the tree. Bell(*k*) denotes the *k*th Bell number, which is the number of unordered partitions of *k *items; these numbers are known to satisfy the bounds ${\left(\frac{k}{e\;\text{In}\;k}\right)}^{k}\;<\;\text{Bell}\left(k\right)<\;{\left(\frac{k}{\text{In}\;k}\right)}^{k}$[7]. Their recursion at a given internal node iterates over every unordered partition of the nonconvex colors, constructing a bipartite graph with edge weightings determined by the sizes of subcolorings of subtrees using those color sets for the set of excluded colors. Applying the Hungarian algorithm to each such graph results in optimal solutions for every possible set of colors to exclude from the subtree at that internal node. Because every unordered partition of the non-convex colors is considered, the algorithm is exponential in *τ*. For the case of general (i.e. not just leaf) colorings, Moran and Snir show that a dynamic program gives an *O *(*n τ d*^*τ*+2^) algorithm. This of course also gives the same bound for leaf colorings.

The work of Moran and Snir was followed up by many authors. For leaf-colored trees, Bachoore and Bodlaender [8] propose a collection of reductions to simplify the problem under investigation. These reductions encode some of the logic of the algorithm presented here, such as that trees that have disjoint color sets can be solved independently. They also use the fact that nonconvexity can be expressed in terms of the crossings of paths connecting leaves of the same color to show that the recoloring problem can be solved in *O*(*n*4^OPT^) time, where OPT is the optimal number of uncolored leaves. Note that this sort of bound is different than those described above, as OPT can get large even when the total number of bad colors is small. The work for the general case culminated in the work of Ponta, Hüffner, and Niedermeier [9], who use the childwise iterative approach to dynamic programing to construct an algorithm of complexity *O*(*n τ *3^τ^).

**Figure 4:**
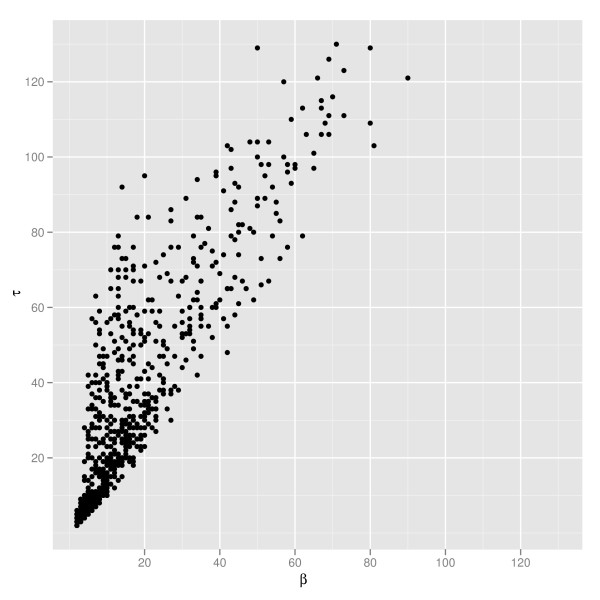
**Local versus global nonconvexity**. The relationship between *β*, a local measure of nonconvexity, and *τ*, a global measure, for our example data set. Each point represents a single phylogenetic tree with taxonomic assignments at a given rank.

For trees built from real data, taxonomic identifiers are not randomly spread across the tree in a uniform fashion. For example, species-level mislabeling will lead to trees that are mostly convex with a couple of outliers, while a horizontal gene transfer will effectively "transplant" one clade into another. In both of these situations there is a non-uniform distribution of taxonomic identifiers across the tree, and nonconvexity in these cases may be local. Indeed, in Figure 4 we show the relationship between the badness *β *and the total number of bad colors *τ *for our example trees, showing that the badness *β *is significantly smaller than the total badness on a collection of phylogenetic trees for non-marker genes. This motivates the search for a fixed parameter tractable algorithm that is exponential in *β *rather than *τ*.

Furthermore, phylogenetics is typically concerned with a setting of trees with small degree. For example, many commonly used phylogenetic inference packages such as RAxML [10] and FastTree [11] only return bifurcating trees; these sorts of programs are the intended source of trees for our algorithm. Even when multifurcations are allowed, the setting of interest is that of degree much smaller than *β *or *τ*, which has ramifications for algorithm choice as described below.

#### Algorithm

In this section we present our algorithm, which makes two improvements over previous work for the parameter regime of interest. First, it restricts attention to cut colors, resulting in an algorithm that is exponential in *β *rather than *τ*. Still, such a complete recursion evaluates many sub-solutions that do not end up being used. Because the problem is NP-hard, we cannot avoid some such evaluation, but we might hope to do better than evaluating everything.

This motivates the second aspect, a branch and bound strategy that can make orders of magnitude improvements in the run time of the algorithm. In order to make the branch and bound algorithm possible, the algorithm enumerates all legal color allocations first, and ranks them according to the upper bound function. By bounding the size of a solution for a given color allocation, we can avoid fully evaluating the sub-solution for that color allocation. A simple way of bounding the size of a solution for a color allocation is the maximal size of the solution when convexity is ignored.

Given a rooted subtree *T' *of *T*, the *root edge *of *T' *in *T *is the edge connecting the root of *T' *to the rest of *T *.

**Definition 6**. *Given a rooted subtree T*' *of T, define κ(T*'*) to be the colors of χ cut by the root edge T*' *as it sits inside T*.

Assume that *T *has been embedded in the plane, and that every internal node has been uniquely labeled. For every such label *i *let *t*(*i*) be the ordered tuple of labels of the nodes directly descendant from *i *in the tree, let *T*(*i*) be the subtree below *i*, and use *κ*(*i*) as shorthand for *κ*(*T*(*i*)). Vector subscript notation will be used to index both *t*(*i*) and the color set *κ*-tuples defined next; i.e. *t*(*i*)*_j _*is the *j*th entry of *t*(*i*).

**Definition 7**. *A *color set *k-*tuple *is an ordered k-tuple of subsets of C. They will be denoted with π*.

These color set *k*-tuples will represent the allocation of colors to subtrees. We will ensure convexity of these color allocations using the following two definitions.

**Definition 8**. *Given a color set k-tuple π*,

$$A\left(\pi \right)\;=\;\underset{i}{⋃}\pi_{i}$$

and

$$B\left(\pi \right)\;=\;\underset{i<j}{⋃}\left(\pi_{i}\;\cap \;\pi_{j}\right).$$

**Definition 9**. *An *almost partition *of Y *⊆ *C is an ordered 2-tuple *(*b, π*) *where b *∈ *C and π is a color set k-tuple such that A*(*π*) = *Y and B*(*π*) ⊆ {*b*}.

These will be the color allocations at a given internal node *x *with color *b*; this definition guarantees convexity locally, such that the *b *color is a legal color for the internal node. As described in the Introduction, we would like to primarily restrict our attention to cut colors, but this requires some attention because not all of the colors that need to be allocated at a given internal node are necessarily cut by the edge above that node. This motivates the following definition, which describes how all of the colors that are cut on the edges around a given internal node *i *are available for allocation at *i *except for those colors that are in *κ*(*i*) but not in *X*.

**Definition 10**. *Given i an internal node index and *X ⊆ *κ*(*i*) *define*

$$G\left(i,\;X\right)\;=\;X\;\cup \;\left(\underset{j\in t\left(i\right)}{⋃}\;\kappa \left(j\right)\;\backslash \;\kappa \left(i\right)\right).$$

*G*(*i, X*) *will be called the *colors in play *for *(*i, X*).

**Definition 11**. *Assume we are given an internal node i, X *⊆ *κ*(*i*), *and c *∈ *C. A *legal color allocation *for *(*i, c, X*) *is an almost partition *(*b, π*) *of G*(*i, X*) *such that*

*1. π_j _*⊆ *κ*(*t*(*i*)*_j_*)

*2. if c *∈ *X then b *= *c*.

*Denote the set of such legal color allocations with *Δ(*i, c, X*), *and let *Δ(*i*) = ∪_*c, X *_Δ(*i, c, X*).

These color allocations are exactly the set of choices that are allowed when developing a subsolution for a cut set *X *such that the color *c *is just above *X*. The first condition ensures that the color allocation for each subtree sits inside the correct set of cut colors. The second condition says that an internal node must take on any color found above and below it.

**Definition 12**. *An implicit subcoloring for T' is a choice of *(*b*(*i*), *π*(*i*)) ∈ Δ(*i*) *for every i *∈ *N*(*T'*) *satisfying the following compatibility property for every k *∈ *t*(*i*):

$$\left(b\left(k\right),\pi \left(k\right)\right)\in \text{Δ}\left(k,b\left(i\right),\pi {\left(i\right)}_{k}\right).$$

That is, the color allocation for every node descending from *i *is a legal color allocation given the choices of *b*(*i*) and *π*(*i*) made at *i*.

As described in the Introduction, an implicit subcoloring defines an actual subcoloring via the implicit subcoloring just proximal to leaf nodes. Indeed, say *t*(*i*)*_j _*is a leaf, and that (*b*(*i*), *π*(*i*)) is the color allocation for internal node *i*. Then *π*(*i*)*_j _*is empty or a single element by definition, and the color for leaf *t*(*i*)*_j _*is used in the subcoloring if *π*(*i*)*_j _*≠ ∅. Every convex subcoloring can be written in this form.

**Proposition 1**. *Implicit subcolorings define convex colorings*.

**Figure 5:**
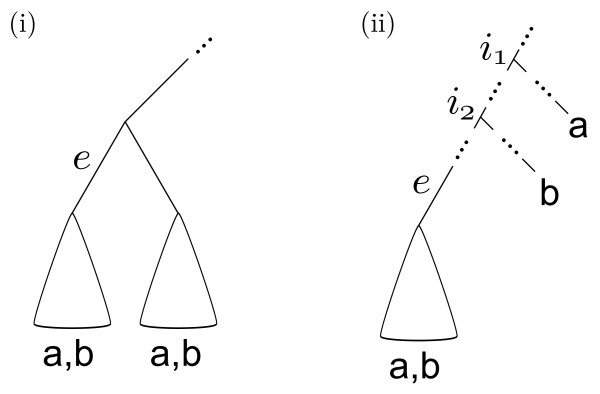
Two potential settings for nonconvexity along an edge *e* in the proof of Proposition 1.

*Proof*. Assume an implicit subcoloring {(*b*(*j*), *π*(*j*))}_*i*∈*N*(*T*)_. Let *χ *be the coloring defined by an implicit subcoloring. If *χ *is not convex, then there is an edge *e *such that $\left|\tilde{\chi }\left(e\right)\right|\;\geq \;2$. Say $\left\{\text{a},\;\text{b}\right\}\;\subseteq \;\tilde{\chi }\left(e\right)$. Without loss of generality, the colors will be positioned as in one of the two cases depicted in Figure 5 (note that the subtrees marked a, b can contain other colors in addition to these). In case (i), |*B*(*π*(*i*))| ≥ 2, contradicting the definition of an almost partition. In case (ii), *b*(*i*_1_) is a by the definition of almost partition because a ∈ *B*(*π*(*i*_1_)). Then *b*(*i*) = a for every *i *between *i*_1 _and *i*_2_, inclusive, by part 2 of Definition 11 and Definition 12. However, b ∈ *B*(*π*(*i*_2_)), contradicting the definition of almost partition. □

With this in mind, we can now speak of the size of an implicit subcoloring as the size of its associated convex subcoloring. The goal, then, is to find the largest implicit subcoloring.

**Theorem 1**. *There is an *O(*n d β*^2 ^2*^d+β ^*(*d*-1)^*dβ*/2^) *complexity algorithm to solve the subcoloring problem for leaf labeled trees*.

*Proof*. For every internal node *i*, define the *question domain Q*(*i*) to be *C *× 2^*κ*(*i*)^. An *answer map *at internal node *i *(resp. *answer size map*) is a map *Y *→ Δ(*i*) (resp. *Y *→ ℕ) for some Y ⊆ *Q*(*i*).

We will fill out an answer map *φ_i _*and an answer size map *ω_i _*as needed at every internal node *i *recursively as follows. For a given *i*, say we are given a question (*c, X*) ∈ *Q*(*i*). If *i *is a leaf, then *φ_i_*(*c, X*) = *X *and *ω_i_*(*c, X*) = |*X*|. Otherwise, say there are *ℓ *descendants of *i*. For each (*b, π*) ∈ Δ(*i, c*, X), find the answers $\varphi_{t{\left(i\right)}_{j}}$ (*b, π_j_*), and their associated $\omega_{t{\left(i\right)}_{j}}$ by recursion. Let

$${\tilde{\omega }}_{i}\left(b,\;\pi \right)\;=\;\underset{1\leq j\leq \ell }{\sum }\omega_{t{\left(i\right)}_{j}}\;\left(b,\;\pi_{j}\right).$$

Let *ω_i_*(*c, X*) be the maximum value of ${\tilde{\omega }}_{i}\left(b,\pi \right)\;\text{for}\;\left(b,\pi \right)\in \text{Δ}\left(i,c,X\right)$, and let *φ_i_*(*c, X*) be the (*b, π*) obtaining this maximum, concluding the recursive step. The result of this recursion after starting at the root with every color for *c *will be a collection of answer maps for every *i*.

These maps define an implicit subcoloring. This can be seen by descending through the tree recursively, using the *φ_i _*to get almost partitions from questions and passing the resulting questions onto subtrees. Specifically, for question (*c, X*) at internal node *i*, let (*b*(*i*), *π*(*i*)): = *φ_i_*(*c, X*) then recur by passing question (*b*(*i*)*_j_, π*(*i*)*_j_*) to $\varphi_{t{\left(i\right)}_{j}}$ for every descendant *j*. Start at the root (call its index *ρ*), pick the color *c_ρ _*maximizing *ω_ρ _*(*c_ρ_*, ∅), and begin the recursion with (*c_ρ_*, ∅).

This subcoloring is maximal by construction. The assertion is clear for 1-leaf trees. Now say that the algorithm finds optimal solutions for all allocations of colors to trees of less than *n *leaves. Then, given a tree on *n *leaves and an allocation of colors to the root, the algorithm tries every legal allocation of those colors to each of the subtrees and taxes the maximum thereof. Because every legal color allocation is tried, and the algorithm finds maximal subcolorings for each of the subtrees, the subcoloring for the entire tree must be maximal.

The computation required for a single internal node is as follows. The number of colors in play is bounded above by *dβ */2, as each color in play must be cut in at least two edges. Thus, for a given question (*c, X*) and color *b *for the internal node, choosing the allocation can take (*d *- 1)^*dβ*/2 ^steps for the colors other than *b*, while deciding where *b *is present can take 2*^d ^*trials. There are at most *β*2*^β ^*choices of question and *dβ*/2 choices of internal node color for a given internal node.

There are *O*(*n*) internal nodes, giving the bound. □

An upper bound for $\tilde{\omega }$ can be used to construct a branch and bound recursion as follows.

**Algorithm 1 **(Branch and bound recursion to find optimal implicit subcoloring). *Assume a function *$\nu_{i}\left(b,\;\pi \right)\;\geq \;{\tilde{\omega }}_{i}\left(b,\;\pi \right)$*for all *(*b, π*) ∈ Δ(*i*). *Proceed as in the proof of Theorem 1, with the following modification. For a given internal node i with c *∈ *C and *X ⊆ *κ*(*i*), *find φ_i_*(*c, X*) *as follows:*

*1. sort the elements *(*b, π*) *of *Δ(*i, c, X*) *in decreasing size with respect to v_i_*.

*2. proceed down this ordered list as follows, starting with q *= 0:

*(a) compute *${\tilde{\omega }}_{i}\left(b,\;\pi \right)\;;\;\;if\;q\;<\;{\tilde{\omega }}_{i}\left(b,\;\pi \right)\;then\;set\;q\;=\;{\tilde{\omega }}_{i}\left(b,\;\pi \right)$

*(b) call the next item in the ordered list *(*b*', *π*'). *If q *≥ *v_i _*(*b*', *π*') *then stop, otherwise recur to (a)*

*3. let φ_i_*(*c, X*) *be the *(*b, π*) *corresponding to q*.

The correctness of this algorithm follows directly from Theorem 1, as the only solutions that are thrown away are strictly sub-optimal.

**Figure 6:**
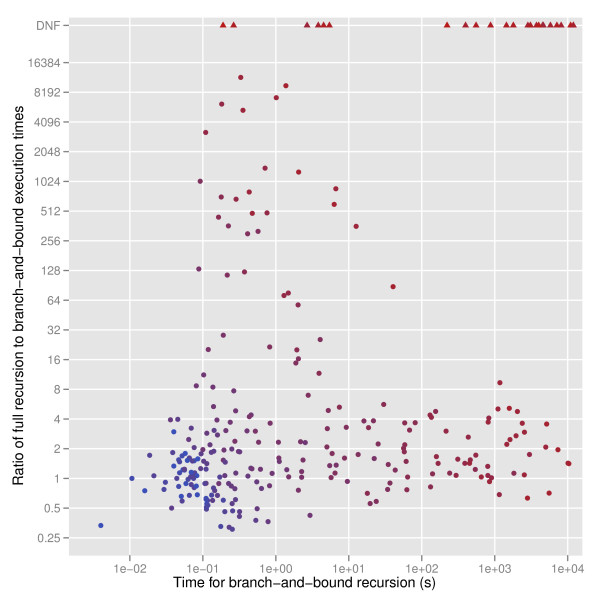
**Runtime comparison.** Runtime comparison between the full recursion (Theorem 1) to the branch and bound (Algorithm 1). "DNF" means that the full recursion did not finish in the time and memory allotted. Symbols colored according to their badness β, ranging from 4 (blue) to 14 (red).

A simple upper bound is the number of leaves that could be used given the restrictions in *π *but ignoring convexity. That is, let ${\bar{v}}_{i}\left(X\right)$ be the number of leaves of *T*(*i*) with colors in *X*. Then define $v_{i}\left(b,\;\pi \right)\;\;=\;\;\sum_{j}{\bar{v}}_{t{\left(i\right)}_{j}}\;\left(\pi_{j}\right)$. This upper bound gives significant improvement in time used over the algorithm in Theorem 1 (Figure 6).

### Computer implementation

The original algorithm described in Theorem 1 and the branch and bound algorithm in Algorithm 1 have been implemented in the rppr binary of the pplacer suite of programs (http://matsen.fhcrc.org/pplacer). The code is in written in OCaml [12], an appropriate choice as it has *O*(log *n*) immutable set operations in the standard library. The input can either be a "reference package" containing both taxonomic and phylogenetic information, or simply a phylogenetic tree along with a comma separated value file specifying the color assignments. Our implementation has been validated using an independent "brute-force" implementation in Python; the two codes return identical results on a testing corpus consisting of all colorings on all trees of three to eight leaves with up to six colors. These trees and results can be downloaded at http://matsen.fhcrc.org/pplacer/data/convexify-validation.tar.gz. The algorithm is invoked via a single command line call, which outputs a list of uncolored taxa for every nonconvex taxonomic rank as well as displaying them on a taxonomically labeled tree by highlighting them in red.

One time saving difference between our implementation and the algorithm described in the previous section is that the computer implementation has a notion of "no color." This is motivated by the fact that in the case that *c *∉ *X *and *B*(*π*) is empty for an internal node *i*, there are a number of colorings of *i *that will provide a convex subtree. By collapsing all of the possible colors into a single "no color" in this case, we gain some savings in time and memory.

The "no color" version of the algorithm can also be used to solve the case of strong convexity described in the Introduction. Specifically, restricting every internal node to have no color except for the internal nodes of subtrees that consist of entirely one color leads to an algorithm for strong convexity. This strong convexity version is available via a command line flag.

The data set used as a test set was a collection of 100 trees built from automatically recruiting sequences via a BLAST search via HMMs built from COG [13] alignments. Taxonomic identifiers for the various ranks were found using the taxtastic software, available at http://github.com/fhcrc/taxtastic. Each trial was run three times and the results averaged; if any of the runs did not finish in 8 hours, exceeded 16 G RAM usage, or encountered a stack overflow, the trial was marked as "DNF." Every trial that completed according to these criteria using the full recursion also completed using the branch-and-bound. Colored trees with badness strictly greater than 14 were excluded from Figure 6, as were trials that did not complete using either algorithm. The full recursion and the branch and bound implementations only differ by a switch that controls if the algorithm terminates early. Trials run on Intel Xeon (X5650) cluster nodes with 48 G of RAM. This test data set is available upon request.

## Taxonomic rooting

Researchers generally like to root phylogenetic trees in a way that the progression along edges from the root to the leaves is one of evolutionary descent. There are a number of ways of achieving this, from using outgroups to using non-reversible models of mutation [14]. However, there has been surprisingly little work on one of the most commonly used informal means of rerooting, which is by using taxonomic classifications. By that we mean looking for a rooting such that the leaf sets of the descendant subtrees each have a single taxonomic classification at the highest taxonomic rank that contains multiple taxonomic identifiers. Here we formalize this process and describe algorithms for finding the taxonomic root or roots.

The work in this section will be based on the following formalization of ranks in taxonomies.

**Definition 13**. *A *rank function *for a set U is a map *rk: 2*^U^***→ **ℕ *such that*

$$\text{max}\left(\text{rk}\left(A\right),\text{rk}\left(B\right)\right)\leq \text{rk}\left(A\cup B\right)$$

*for all A and B in *2*^U^*

It follows immediately that rk(*A*) ≤ rk(*B*) when *A *⊆ *B *∈ 2*^U^*. By an abuse of notation, we also let rk(*T*) signify rk(*L*(*T*)) for a (sub)tree *T *with leaf set in the domain of the rank function. From a taxonomic perspective, rk(*U*) will represent the rank of the most specific taxonomic classification containing all of the taxonomic labels in *U*. For this section, *a taxonomically labeled phylogenetic tree *is one for which we have a rank function on the leaves.

Given *x *a node of *T*, let Ψ(*x*; *T*) represent the set of trees obtained by rooting *T *at *x *and deleting *x *and its incident edges from *T *.

**Definition 14**. *Define the *subrank subrk(*x*; *T*) *to be *max_*S*∈Ψ(*x*;*T*) _rk(*S*), *the maximum rank of the subtrees of T when rooted at x. We will say x is a *delicate taxonomic root *of T if*

$$\text{subrk}\left(x;\;T\right)\;=\;\underset{y\in N\left(T\right)}{\text{min}}\;\text{subrk}\left(y;\;T\right).$$

This definition formalizes an intuitive definition of taxonomic root. For example, imagine that we have a tree with the three domains of cellular life in three distinct subtrees: Bacteria, Archaea, and Eukaryota; call the internal node that sits between these subtrees *x*. The subrank of *x *is domain. Any other internal node will contain some of each of the domains, and thus will have rank strictly higher than domain. In this case, *x *is the unique taxonomic root.

However, if the tree is not convex at the subrank of the delicate taxonomic root then every internal node will be a delicate taxonomic root; thus the "delicate" terminology. Indeed, assume an internal node *y *and *A, B *∈ Ψ(*y*; *T*) such that *a*_1_, *a*_2 _⊆ *L*(*A*), *b*_1_, *b*_2 _⊆ *L*(*B*), and subrk(*a*_1_, *a*_2_) = subrk(*b*_1_, *b*_2_) = subrk(*T*). Then for any rooting, there must exist a subtree containing either {*a*_1_, *a*_2_} or {*b*_1_, *b*_2_}, and the subrank must be equal to that of *T*.

We now develop a more robust definition of taxonomic root, which will require several definitions. The edges of the tree will be thought of as unordered pairs {*x, y*} of nodes.

**Definition 15**. *An arrow on an edge *{*x, y*} *is an ordered pair of nodes *(*x, y*). *The first node of the pair is called the origin of the arrow, and the second is called the direction*.

**Definition 16**. *An *arrow tree (T,A) is an ordered pair consisting of a tree T and a set of arrows A *on the edges of T. A *complete arrow *tree is an arrow tree such that for every node x of the tree there is some arrow in *A *with origin x*.

Note that (*x, y*) and (*y, x*) may both be part of an arrow set for a tree with an edge {*x, y*}. We will use "pointing towards" and "pointing away" in their usual senses as they relate to arrows in the real world.

**Definition 17**. *The *induced arrow tree (T,A)*for a tree T and a rank function *rk *is a complete arrow tree defined as follows. For a given internal node x *∈ *N*(*T*), *say *{*S*_1_, · · ·, *S_n_*} = Ψ(*x*; *T*) *and let r_i _*= rk(*S_i_*) *for *1 ≤ *i *≤ *n. Assume without loss of generality that r*_1 _≤ *r*_2 _≤ · · · ≤ *r_n_. There is some minimal *1 ≤ *j *≤ *n such that r_j _*= · · · = *r_n_. Let ***A***_x _be*

$$\left\{\left(x,y\right)|y\;is\;the\;root\;of\;one\;of\;S_{j},\cdots \;,S_{n}\right\}.$$

*Then *A *is the union of the *$A_{x}$*for all nodes x along with the set of *(*x, y*) *where x is a leaf and y is adjacent to x*.

Intuitively, induced arrows point towards potential taxonomic roots.

**Lemma 1**. *Say *(T,A)*is an induced arrow tree for a rank function *rk, *and that *{*x*, y} *and *{*y, z*} *are adjacent edges of T. If *(y,z)∈A*then *(x,y)∈A.

**Figure 7:**
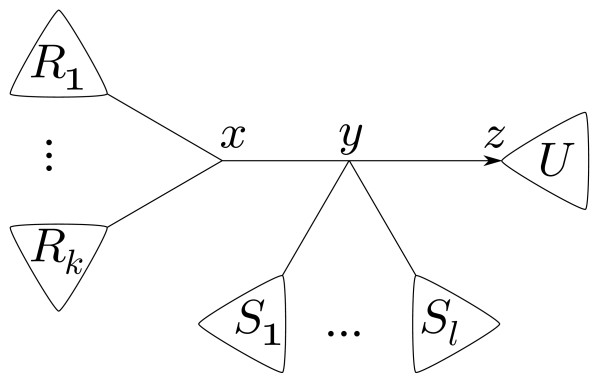
Illustration of Lemma 1.

*Proof*. When *x *is a leaf, (x,y)∈A is automatic, thus assume it is not. Using terminology from Figure 7, because (y,z)∈A,

$$\text{rk}\left(R_{1}\;\cup \;\cdot \;\cdot \;\cdot \;\cup \;R_{k}\right)\;\leq \;\text{rk}\left(U\right).$$

This implies that

$$\text{rk}\left(R_{i}\right)\;\leq \;\text{rk}\;\left(U\right)\;\leq \;\text{rk}\left(S_{1}\;\cup \;\cdot \;\cdot \;\cdot \;\cup \;S_{\ell }\;\cup \;U\right)$$

and thus that (x,y)∈A. □

Induction on the edges of a path shows the following:

**Corollary 1**. *Say *(T,A)*is an induced arrow tree, and that *{*u, v*} *and *{*x, y*} *are edges of T such that the path from u to y contains both v and x. If *(x,y)∈A*then *(u,v)∈A. □

Informally, this corollary says that any time there is an arrow on edge *e*_2 _pointing away from edge *e*_1_, that there must be an arrow on *e*_1 _pointing towards *e*_2_.

**Definition 18**. *A multi arrow node (MAN) for a taxonomically labeled tree is a node x such that there are two or more arrows in the induced arrow tree with x as an origin*.

**Proposition 2**. *Say *(T,A)*is an induced arrow tree. If node x is a MAN then for any node y there must be an arrow in *A *with origin y pointing towards x*.

*Proof*. Since *x *is a MAN, there must be at least one arrow in A  with origin *x *pointing away from *y*. This implies the proposition by Corollary 1. □

Now imagine that *x *and *z *are two MANs, and *y *is on the path between *x *and *z*. By the above proposition, there must be arrows with origin *y *pointing towards both *x *and *z*, showing that *y *will be a MAN. Thus:

**Proposition 3**. *MANs form a convex set in the tree*. □

**Definition 19**. *An edge *{*x, y*} *is a *bi-arrow edge *of an arrow set A if *(*x, y*) *and *(*y, x*) *are in A*.

**Proposition 4**. *If the set of MANs is empty, then there is exactly one bi-arrow edge*.

*Proof*. First note that there cannot be two or more bi-arrow edges when the set of MANs is empty; in that case by Corollary 1 there would have to be a MAN between them. Now assume there are no bi-arrow edges. Since the set of MANs is empty, then for every leaf of the tree the sequence of nodes determined by following arrows is well defined. Note that the arrow on every leaf is pointing into the interior of the tree, and thus the sequence of nodes starting from an arbitrary leaf cannot hit another leaf. Therefore the sequence of nodes must backtrack somewhere, contradicting that there are no bi-arrow edges. □

**Definition 20**. *Assume a taxonomically labeled tree T. If there is at least one MAN then define the set of taxonomic roots to be the set of MANs. Otherwise define it to be the set of nodes of the bi-arrow edge*.

Let diam(*T*) be the node-diameter of *T*, i.e. the number of steps from edge to edge required to traverse the tree. Because every arrow with a non-root origin points in the direction of the taxonomic roots:

**Proposition 5**. *A taxonomic root for a tree T with n leaves can be found in at most diam*(*T*) *steps*. □

### Computer implementation

Taxonomic rerooting has been implemented in the rppr binary of the pplacer suite of programs (http://matsen.fhcrc.org/pplacer). However, rather than finding all possible taxonomic roots as described above, the program reports one of the roots after applying the maximal subcoloring algorithm as described in the previous section to the highest multiply occupied taxonomic rank. Such a root is the closest approximation to the one "best" taxonomic root in the presence of nonconvexity.

## Conclusions and future work

We have formalized the question of describing the discordance of a phylogenetic tree with its taxonomic classifications in terms of a convex subcoloring problem previously described in the literature. This coloring problem has some elegant solutions for the general case, but the parameter regime of interest here consists of trees of small degree and local nonconvexity. These considerations motivate a solution that solves a given recursion for as few "questions" as possible. The first component of this is to restrict attention to cut colors, resulting in a smaller base for the exponential complexity (Figure 4). The second is a branch and bound algorithm that gives a significant improvement in runtime compared to the algorithm in Theorem 1 (Figure 6). To enable this the *φ_i _*are only built up "upon demand," that is, when a given question is asked. The implementation described here is the first of which we are aware, and certainly the first that conveniently integrates with taxonomic annotation.

We also develop the first formalism for taxonomic rooting of phylogenetic trees, show that the obvious definition is useless in the presence of nonconvexity, and develop a more robust definition. This version can be found in time linear in the diameter in the tree.

We are currently developing a computational pipeline to find misclassified sequences in public databases using these algorithms. We are also using these algorithms together to develop a collection of automatically curated "reference packages" that bring together taxonomic and phylogenetic for the purposes of environmental short read classification, visualization, and comparison.

## Competing interests

The authors declare that they have no competing interests.

## Authors' contributions

FAM conceived of the project, designed the algorithms, and wrote the paper. AG designed the algorithms and implemented the algorithms and validations. Both authors read and approved the final manuscript.
